# Supplementary material for: OmrA sRNA inhibits translation of phosphoenolpyruvate carboxylase to impair TCA-cycle flux
Source: Nucleic Acids Res. 2026 Aug 14;54(15):gkag804. doi: 10.1093/nar/gkag804 (PMC13473878; doi:10.1093/nar/gkag804)
Supplement: Supplementary file 1 [file gkag804_supplemental_files.zip › Supplementary data_revised_v2.pdf]

Supplementary Data

OmrA sRNA Inhibits Translation of Phosphoenolpyruvate Carboxylase to Impair TCA-Cycle Flux

Thomas Stenum*[1], Kim Boi Le Huyen*[1], Jonas Kjellin[2,3], Sanna Koskiniemi[1], E. Gerhart H. Wagner[1], and Erik Holmqvist[1]

Affiliations:

[1]Department of Cell and Molecular Biology, Biomedical Centre, Uppsala University, Uppsala, Sweden

[2]Department of Medical Sciences, SNP&SEQ Technology Platform, Uppsala University, Uppsala, Sweden

[3]Science for Life Laboratory, Uppsala University, Uppsala, Sweden

*These authors contributed equally to this work.

To whom correspondence should be addressed:
E-mail: erik.holmqvist@icm.uu.se; gerhart.wagner@icm.uu.se

Keywords: sRNA, RNA toxicity, TCA cycle, Hfq, post-transcriptional control

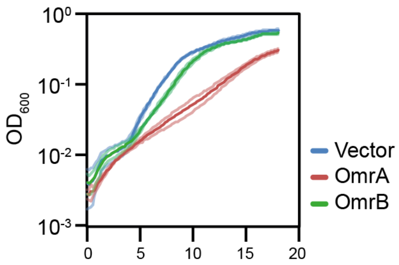

Figure S1. *E. coli* MG1655 Δ*omrAB* harboring an empty vector, pOmrA, or OmrB, was grown in M9-glucose media in a 96 well plate. Bright colored lines: mean growth rate. Faint lines: standard deviation based on four biological replicates.

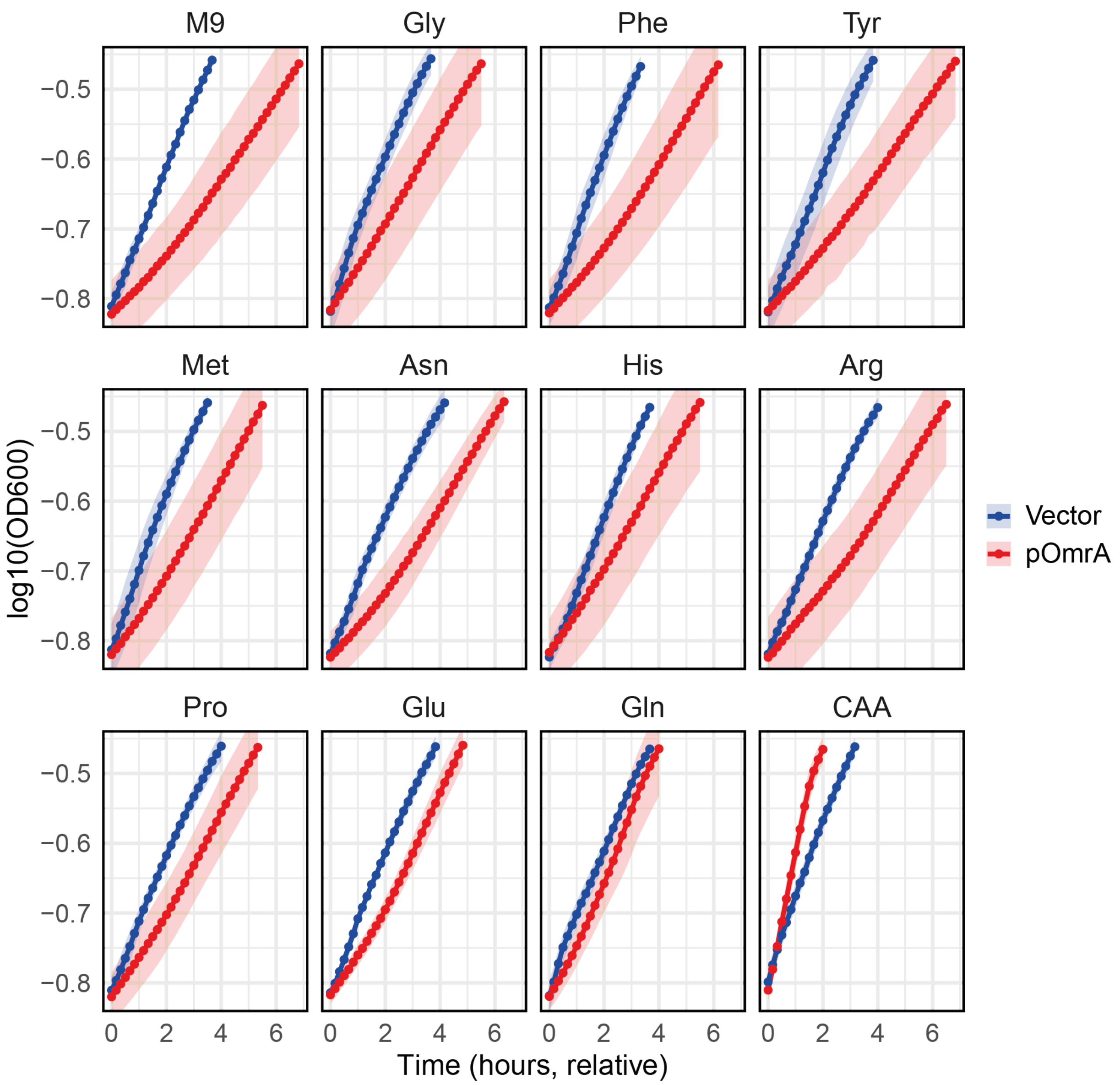

Figure S2. *E. coli* MG1655 Δ*omrAB* harboring an empty vector or pOmrA was grown in M9-glucose media supplemented with the indicated amino acids in a 96 well plate. The exponential growth phase is shown.

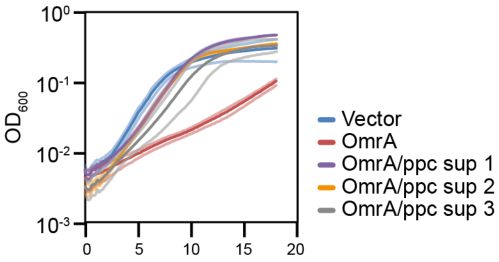

Figure S3. Growth of suppressor strains harboring mutations in *ppc* upon OmrA overexpression. Bright colored lines: mean growth rate. Faint lines: standard deviation based on four biological replicates.

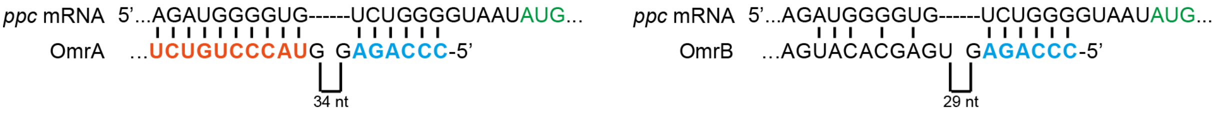


```
OmrA    CCCAGAGGTATTGATTGGTGAGATTATTCGGTACGCTCTTCGTACCCTGTCTCTTGCACCAACCTGCGCGGATGCGCAGGTTTTTTTT
OmrB    CCCAGAGGTATTGATAGGTGAAGTCA------ACTTCGGGTTGAGCACATGAATTACACCAGCCTGCGCAGATGCGCAGGTTTTTTTT
        *************** *****..*.*      ** ..   .  * * ..*   **.*****.*******.*****************
```

Figure S4. Predicted base-pairing between OmrA or OmrB and the *ppc* 5'UTR (top panels). Alignment indicating sequence deviations in the middle regions of the two sRNAs.

Table S1. Oligos used in this study

Name	Description	Sequence (5'-3')
EHO-1006	Fwd for PCR of pZE12-luc blunt (pLlacO-1)	GTTTTTATGCATGTGCCACCTGACGTCTAAGAAAC
EHO-1122	Rev for PCR of pZE12-luc blunt (pLlacO-1)	CGGCGGATTTGTCCTACT
EHO-827	Fwd for colony PCR on pXG10-SF. Used for plasmid ppc-GFP sequencing.	TGGGATATATCAACGGTGGT
EHO-828	Rev for colony PCR on pXG10-SF. Used for plasmid ppc-GFP sequencing.	GAATTGGGACAACTCCAGTG
EHO-2287	Fwd for creating a translational fusion of MG1655 ppc to GFP-SF in pXG10-SF, including the whole 5'UTR and the first 20 codons of the ORF. With NsiI site and 5 nt overhang	GTTTTATGCATGACGACGAAAAGCAAAGCC
EHO-2288	Rev for creating a translational fusion of MG1655 ppc to GFP-SF in pXG10-SF, including the whole 5'UTR and the first 20 codons of the ORF. With NheI site and 5 nt overhang	GTTTTGCTAGCTCCCAGCACTTTGCCGAG
EHO-2289	Fwd for introducing mutation 1 (position -10:C->T) in the ppc-GFP translational fusion plasmid	TGGGGTGTTTGGGGTAATATGAACGAACAATATTCC
EHO-2290	Rev for introducing mutation 1 (position -10:C->T) in the ppc-GFP translational fusion plasmid	TTACCCCAAACACCCCATCTTATCGTTTGATAGCCC
EHO-2291	Fwd for introducing mutation 2 (position -8: G->T) in the ppc-GFP translational fusion plasmid	GGGTGTCTTGGGTAATATGAACGAACAATATTCCGC
EHO-2292	Rev for introducing mutation 2 (position -8: G->T) in the ppc-GFP translational fusion plasmid	TATTACCCAAGACACCCCATCTTATCGTTTGATAGC
EHO-2293	Fwd for introducing mutation 3 (position -5: G->C) in the ppc-GFP translational fusion plasmid	TGTCTGGGCTAATATGAACGAACAATATTCCGCATT
EHO-2294	Rev for introducing mutation 3 (position -5: G->C) in the ppc-GFP translational fusion plasmid	TCATATTAGCCCAGACACCCCATCTTATCGTTTGAT
EHO-2307	Fwd for C-terminal 3xFLAG tagging of ppc, containing region upstream of stop codon of ppc (in capital), and homologous region of 3x-FLAG-FRT-KmR-FRT from pSUB11 (in non-capital).	CACTATTGCCGGGATTGCGGCAGGTATGCGTAATACCGGCgactacaaagaccatgacgg
EHO-2308	Rev for C-terminal 3xFLAG tagging of ppc, containing region upstream of stop codon of ppc (in capital), and homologous region of 3x-FLAG-FRT-KmR-FRT from pSUB11 (in non-capital).	ACCCTCGCGCAAAAGCACGAGGGTTTGCAGAAGAGGAAGAcatatgaatatcctccttag
EHO-2309	Fwd to MG1655 ORF of ppc. Used for PCR screen of 3xFLAG insertion on C-term of ppc	GAAGACATCAAAGTGGTGCTG
EHO-2310	Rev to region downstream of MG1655 ppc ORF. Used for PCR screen of 3xFLAG insertion on C-terminal of ppc	AATGGCCTGTAGCAGAGTAGA
EHO-2312	Fwd for T7 for ppc gene. Used for in vitro transcription.	gaaattaatacgactcactataGGACGACGAAAAGCAAAGCCCGAG
EHO-2313	Rev for T7 for ppc gene. Used for in vitro transcription.	TACTGACATTACTACGCAATGCGGAA
EHO-2470	Rev for toeprint assay of ppc gene	TTCTCCCAACGCATCCTTGATGGTTT

EHO-2479	Fwd for introducing mutation ppc M1*(position -9: GUC -> CUC) in the ppc-GFP translational fusion plasmid	GTCTCGGGTAATATGAACGAACAATATTC
EHO-2480	Rev for introducing mutation ppc M1*(position -9: GUC -> CUC) in the ppc-GFP translational fusion plasmid	ACCCGAGACACCCCATCTTATC
EHO-2234	Fwd T7-arcZ, anneal to EHO-2235	gtttttttaatacgactcactataggTTTCCCTGGTGTTGGCGCAGTATTCGCGCACCCCGGTCAAACCGGGGTCATTTTTT
EHO-2235	Rev T7-arcZ, anneal to EHO-2234	AAAAAATGACCCCGGTTTGACCGGGGTGCGCGAATACTGCGCCAACACCAGGGAAAcctatagtgagtcgtattaaaaaaaac
EHO-2370	Fwd for introducing mutation omrA M3 (position 43: TAC -> GTG) into pEH67	TCGGTGCCTGTCTCTTGC
EHO-2371	Rev for introducing mutation omrA M3 (position 43: TAC -> GTG) into pEH67	AGGCACCGAAGAGCGTAC
EHO-245	Fwd for FRT-Tet-FRT ampli with omrB upstream homology (in capital), for omrAB deletion with lambda red	CCCGTTGGTTCAAGGGGTGGCGTGTTTTCATCGTGGGAATgaattcgagctcggtacccg
EHO-246	Rev for FRT-Tet-FRT ampli with omrA homology (in capital), for omrAB deletion with lambda red	CCTGCGCATCCGCGCAGGTTGGTGCAAGAGACAGGGTACgctatgaccatgattacgcc
EHO-247	Fwd upstream of omrAB deletion, 5' junction PCR	CTTAGTGGGTAAACGCTTACATTC
EHO-249	Rev downstream of omrAB deletion, 3' junction PCR	CAACTCCCTTTGCTCTGATTCAG
EHO-2528	For T7-Hammerhead-omrA M1, anneal to EHO-2529, fill-in	gaaattaatacgactcactataGGGAGATGGGCTGATGAGTCCGTGAGGACGAAACGGTACCCGGTACCGTCCCGAGAGGTATTGATTGG
EHO-2529	Rev T7-Hammerhead-omrA M1, anneal to EHO-2528, fill-in	AAAAAAAACCTGCGCATCCGCGCAGGTTGGTGCAAGAGACAGGGTACGAAGAGCGTACCGAATAATCTCACCAATCAATACCTCTCGGGA
EHO-406	OmrA northern probe	GAGACAGGGTACGAAGAGCGTACCGAATAATCTCACC
EHO-407	OmrB northern probe	GTGTAATTCATGTGCTCAACCCGAAGTTGACTTCACC
EHO-867	tmRNA northern probe	TGGTGGAGCTGGCGGGAGTT

Table S2. Bacterial strains used in this study

**Strain number**	**Name**	**Genotype**	**Plasmid**	**Reference**
	E. coli TOP10	F- mcrA Δ(mrr-hsdRMS-mcrBC) Φ80lacZΔM15 Δ lacX74 recA1 araD139 Δ(araleu)7697 galU galK rpsL (StrR) endA1 nupG		
EHS-4092	E. coli TOP10	F- mcrA Δ(mrr-hsdRMS-mcrBC) Φ80lacZΔM15 Δ lacX74 recA1 araD139 Δ(araleu)7697 galU galK rpsL (StrR) endA1 nupG	pTSS58	this study
EHS-4102	E. coli TOP10	F- mcrA Δ(mrr-hsdRMS-mcrBC) Φ80lacZΔM15 Δ lacX74 recA1 araD139 Δ(araleu)7697 galU galK rpsL (StrR) endA1 nupG	pTSS59	this study
EHS-4103	E. coli TOP10	F- mcrA Δ(mrr-hsdRMS-mcrBC) Φ80lacZΔM15 Δ lacX74 recA1 araD139 Δ(araleu)7697 galU galK rpsL (StrR) endA1 nupG	pTSS60	this study
EHS-4104	E. coli TOP10	F- mcrA Δ(mrr-hsdRMS-mcrBC) Φ80lacZΔM15 Δ lacX74 recA1 araD139 Δ(araleu)7697 galU galK rpsL (StrR) endA1 nupG	pTSS61	this study

EHS-4145	E. coli TOP10	F- mcrA Δ(mrr-hsdRMS-mcrBC) Φ80lacZΔM15 Δ lacX74 recA1 araD139<br>Δ(araleu)7697 galU galK rpsL (StrR) endA1 nupG	pKB009	this study
KBS-0100	E. coli TOP10	F- mcrA Δ(mrr-hsdRMS-mcrBC) Φ80lacZΔM15 Δ lacX74 recA1 araD139<br>Δ(araleu)7697 galU galK rpsL (StrR) endA1 nupG	pKB013	this study
EHS-3164	MG1655	Δ *omrAB*::FRT-TetR-FRT	pJV300	this study
EHS-3165	MG1655	Δ *omrAB*::FRT-TetR-FRT	pEH67	this study
EHS-3166	MG1655	Δ *omrAB*::FRT-TetR-FRT	pEH68	this study
EHS-3833	MG1655	Δ *omrAB*::FRT-FRT	pJV300	this study
EHS-3834	MG1655	Δ *omrAB*::FRT-FRT	pEH67	this study
EHS-3835	MG1655	Δ *omrAB*::FRT-FRT	pEH68	this study
EHS-4020	MC4100 relA-		pJV300	(1)
EHS-4021	MC4100 relA-	*hfq-F42A*	pJV300	(1)
EHS-4022	MC4100 relA-	*hfq-Y25D*	pJV300	(1)
EHS-4023	MC4100 relA-	*hfq-R16A*	pJV300	(1)
EHS-4026	MC4100 relA-		pEH67	(1)
EHS-4027	MC4100 relA-	*hfq-F42A*	pEH67	(1)
EHS-4028	MC4100 relA-	*hfq-Y25D*	pEH67	(1)
EHS-4029	MC4100 relA-	*hfq-R16A*	pEH67	(1)
EHS-4093	MG1655	Δ *omrAB*::FRT-FRT	pXG-0, pJV300	this study
EHS-4095	MG1655	Δ *omrAB*::FRT-FRT	pTSS58, pJV300	this study
EHS-4098	MG1655	Δ *omrAB*::FRT-FRT	pTSS58, pEH67	this study
KBS-0081	MG1655	Δ *omrAB*::FRT-FRT	pTSS58, pEH171	this study
KBS-0082	MG1655	Δ *omrAB*::FRT-FRT	pTSS58, pEH80	this study
KBS-0085	MG1655	Δ *omrAB*::FRT-FRT	pTSS58, pKB009	this study
KBS-0106	MG1655	Δ *omrAB*::FRT-FRT	pKB013, pJV300	this study
KBS-0107	MG1655	Δ *omrAB*::FRT-FRT	pKB013, pEH67	this study
KBS-0108	MG1655	Δ *omrAB*::FRT-FRT	pKB013, pEH171	this study
EHS-4242	MG1655	*ppc*-3xFLAG (C-terminal)		this study
EHS-4243	MG1655	Δ *omrAB*::FRT-FRT *ppc*-3xFLAG (C-terminal)		this study
KBS-0165	MG1655	Δ *omrAB*::FRT-FRT *ppc*-3xFLAG (C-terminal)	pJV300	this study
KBS-0166	MG1655	Δ *omrAB*::FRT-FRT *ppc*-3xFLAG (C-terminal)	pEH67	this study
KBS-0167	MG1655	Δ *omrAB*::FRT-FRT *ppc*-3xFLAG (C-terminal)	pEH171	this study
KBS-0168	MG1655	Δ *omrAB*::FRT-FRT *ppc*-3xFLAG (C-terminal)	pEH68	this study
KBS-0169	MG1655	Δ *omrAB*::FRT-FRT *ppc*-3xFLAG (C-terminal)	pEH172	this study

Table S3. Plasmids used in this study

Name	Description	Marker	Reference
pJV300	pZE12-luc based control plasmid, -1 site religated to second position of XbaI site, destroys XbaI site (colE1, Amp), PLlacO promoter reads into rrnB terminator	Amp	(2)
pXG-0	Control plasmid for cellular autofluorescence	Cm	(3)
pXG10-SF	pXG-10 derivative with sfgfp, for translational sfGFP fusions	Cm	(4)
pSIM5-Tet	λ red expression plasmid	Tet	(5)
pTSS58	*ppc-gfp* translational fusion. The leader of ppc (from TSS until 20 codons into the gene) was cloned inframe with the GFP in pXG10-SF using NsiI and NheI RE sites.	Cm	this study
pTSS59	*ppc M1-gfp* translational fusion. The leader of ppc with a C->T mutation at the -10 position (from TSS until 20 codons into the gene) was cloned inframe with the GFP in pXG10-SF using NsiI and NheI RE sites.	Cm	this study
pTSS60	*ppc M2-gfp* translational fusion. The leader of ppc with a G->T mutation at the -8 position (from TSS until 20 codons into the gene) was cloned inframe with the GFP in pXG10-SF using NsiI and NheI RE sites.	Cm	this study
pTSS61	*ppc M3-gfp* translational fusion. The leader of ppc with a G->C mutation at the -5 position (from TSS until 20 codons into the gene) was cloned inframe with the GFP in pXG10-SF using NsiI and NheI RE sites.	Cm	this study
pKB013	based on pTSS58, with *ppc M1** compensatory (EHO-2479/2480) to OmrA M1	Cm	this study
pEH67	OmrA cloned in pZE12-luc blunt (pLlacO-1)	Amp	(6)
pEH68	OmrB cloned in pZE12-luc blunt (pLlacO-1)/XbaI	Amp	(6)
pEH171	based on pEH67, with OmrA M1 (EHO-2370/2371) mutated position 3-C -> G	Amp	(7)
pEH80	based on pEH67, with OmrA M2 mutated position1-2: CC -> GG	Amp	(7)
pKB009	based on pEH67, with OmrA M3 (EHO-2370/2371) mutated position 43-TAC -> GTG	Amp	this study

References

1. Zhang,A., Schu,D.J., Tjaden,B.C., Storz,G. and Gottesman,S. (2013) Mutations in interaction surfaces differentially impact E. coli Hfq association with small RNAs and their mRNA targets. *Journal of Molecular Biology*, **425**, 3678–3697.

2. Sittka,A., Pfeiffer,V., Tedin,K. and Vogel,J. (2007) The RNA chaperone Hfq is essential for the virulence of Salmonella typhimurium. *Mol Microbiol*, **63**, 193–217.

3. Urban,J.H. and Vogel,J. (2007) Translational control and target recognition by Escherichia coli small RNAs in vivo. *Nucleic Acids Research*, **35**, 1018–1037.

4. Corcoran,C.P., Podkaminski,D., Papenfort,K., Urban,J.H., Hinton,J.C.D. and Vogel,J. (2012) Superfolder GFP reporters validate diverse new mRNA targets of the classic porin regulator, MicF RNA. *Molecular Microbiology*, **84**, 428–445.

5. Datta,S., Costantino,N. and Court,D.L. (2006) A set of recombineering plasmids for gram-negative bacteria. *Gene*, **379**, 109–115.

6. Holmqvist,E., Reimegård,J., Sterk,M., Grantcharova,N., Römling,U. and Wagner,E.G.H. (2010) Two antisense RNAs target the transcriptional regulator CsgD to inhibit curli synthesis. *The EMBO Journal*, **29**, 1840–1850.

7. Romilly,C., Hoekzema,M., Holmqvist,E. and Wagner,E.G.H. (2020) Small RNAs OmrA and OmrB promote class III flagellar gene expression by inhibiting the synthesis of anti-Sigma factor FlgM. *RNA Biology*, **17**, 872–880.
